# Interferon Inducible IFI16 Expression in p16 Positive Squamous Cell Carcinoma of the Oropharynx

**DOI:** 10.1155/2013/263271

**Published:** 2013-07-11

**Authors:** Moriyasu Yamauchi, Takafumi Nakano, Torahiko Nakashima, Ryuji Yasumatsu, Kazuki Hashimoto, Satoshi Toh, Hideki Shiratsuchi, Yoshinao Oda, Shizuo Komune

**Affiliations:** ^1^Department of Otorhinolaryngology, Head and Neck Surgery, Graduate School of Medical Sciences, Kyushu University, Fukuoka 812-8582, Japan; ^2^Department of Anatomic Pathology, Graduate School of Medical Sciences, Kyushu University, Fukuoka 812-8582, Japan

## Abstract

Human-papillomavirus- (HPV-) positive oropharyngeal squamous cell carcinomas (OPSCC) are reported to be more responsive to treatment and to be related to a favorable prognosis compared with non-HPV carcinomas. However, the molecular basis of the responsiveness is unclear. Interferon inducible IFI16, which is implicated in the control of cell growth, apoptosis, angiogenesis, and immunomodulation in various types of cancers, is reported to be frequently expressed in the HPV-positive head and neck SCC and to correlate with a better prognosis. In this study, we hypothesized that HPV related OPSCC expresses IFI16 resulting in favorable prognosis. To clarify the relationship between the prognosis of HPV related OPSCC patients and IFI16 status, we examined immunohistologically the pretreatment specimens of OPSCC for the expression of p16 as a surrogate marker of HPV infection and IFI16. We could not show that the expression of IFI16 is associated with that of p16. There was no significant difference in the survival rate between IFI16 positive and negative groups. Patients with p16 negative tumor exhibited worse survival rate regardless of IFI16 status. In this limited case series, we could not conclude that IFI16 expression is altered in p16 positive OPSCC and that it would be a new predictive marker or a useful therapeutic tool.

## 1. Introduction

Mucosal human papillomavirus (HPV) infections are well known to associate with invasive carcinomas of cervix and anogenital region. Recently, HPV has been found to be etiologically involved in 20% to 25% of head and neck squamous cell carcinoma (SCC), mostly in the oropharynx [1]. HPV-positive oropharyngeal cancers are reported to be more responsive to treatment and to show a favorable prognosis compared with non-HPV carcinomas [2]. However, the molecular basis of the responsiveness is unclear.

IFI16 is a member of the Interferon- (IFN-) inducible HIN200 gene family which can be induced by IFN stimulation followed by several intracellular signaling cascades. The family includes a group of human (IFI16, IFIX, MNDA, and AIM2) and mouse (Ifi202a, Ifi202b, Ifi203, Ifi204, and D3/Ifi205) genes and they mediate the necessary biological responses [3]. These proteins share a partially conserved repeat of 200 amino acid residues (the HIN-200 domain) towards the C-terminus, which allows these proteins to bind dsDNA. Most p200-family proteins also contain a homotypic protein-protein interaction PYRIN domain (PYD) in the N-terminus [4]. IFI16 is implicated in the control of cell growth, apoptosis, angiogenesis, and immunomodulation in various types of cancers [5]. Several reports show that IFI16 interacts with p53 and enhances the transcriptional activation function of p53 [3, 6]. IFI16 is also indicated to bind to the hypophosphorylated form of the retinoblastoma protein (pRb) resulting in cell cycle arrest through inhibition of the E2F1-mediated transcription [7].

For patients with resectable head and neck SCC treatment with surgery and (chemo-)radiotherapy is considered as a standard approach. In the surgical treatment, radical resection is preferable for the management of disease, but organ preservation is also desirable for the cosmetic and functional purposes. Balancing these two conflicting goals is difficult. Radiotherapy plays an integral part of the treatment strategy for the organ preservation. Although radiotherapy accompanies several adverse events, not all the patients achieve the complete response. It would be very helpful to determine the therapeutic strategy if radiation sensitivity was predictable. Furthermore, it would be a powerful therapeutic tool, if possible, to induce molecules which can sensitize tumors to radiotherapy.

Azzimonti et al. reported that IFI16 is frequently expressed in the HPV-positive head and neck SCC and correlates with a better prognosis [8]. One possibility is that in the HPV related oropharyngeal SCC (OPSCC), the expression of IFI16 protein is upregulated and contributing to the inhibition of tumor progression. If IFI16 was acting as a tumor repressor in the HPV related OPSCC, it would be a new predictive marker or induction of IFI16 would be a new therapeutic arm in IFI16 negative SCC. In this study, to clarify the relationship between a better prognosis in HPV associated OPSCC patients and IFI16 status, we examined the pretreatment specimen of OPSCC immunohistologically for the expression of p16 and IFI16 protein.

## 2. Materials and Methods

### 2.1. Patients and Tumor Specimens

Patients with OPSCC arising from tonsil or base of tongue treated at the Kyushu University Hospital were identified through the surgical pathology files and tumor registry. Clinical information was collected from clinical record. The clinical staging and identification of the anatomical site of the tumors were based on the International Union for Cancer Control (UICC) TNM classification of malignant tumors, the 7th edition.

### 2.2. Immunohistochemistry

Immunohistochemical evaluation was performed as reported previously [9]. Briefly, 4 *μ*m thick sections were cut onto 2% organosilane-coated slides. The deparaffinized and rehydrated slides were then digested in 0.05% trypsin for 10 min. After incubation in methanol containing 0.3% hydrogen peroxide for 30 min to block endogenous peroxidase activity, primary antibody was applied to sections for 15 h at 4°C. The primary antibody used was mouse anti-human IFI-16 (sc-8023) antibody (purchased from Santa Cruz Biotechnology) and mouse anti-human p16 (13251A) antibody (purchased from Pharmingen). The streptavidin biotin peroxidase method (Histofine MAX-PO kit; Nichirei, Japan) was used for detection, employing 3,3′-diaminobenzidine (DAB) as the chromogen. The sections were counterstained slightly with hematoxylin. The evaluation was performed blind to clinical information. All series included positive and negative controls.

### 2.3. Statistical Analysis

Statistical analyses were performed using the Mann-Whitney *U* test. The disease-specific survival rate was calculated by the Kaplan-Meier method. The significance of differences of survival plots was analyzed by the log-rank test. Differences with a *P* value < 0.05 were considered to be significant.

## 3. Results

### 3.1. Characterization of Patients

The distribution of 22 tumors by anatomical site was as follows: 20 tumors in the tonsil and 2 in the base of tongue (Table 1). The age of the patients at diagnosis ranged from 37 to 84 years (mean 61 years). Twenty patients were male and 2 were female. The followup of the patients ranged from 2 to 108 months (mean 34.6 months).

The result of the clinical staging was as follows: T1 18% (4/22); T2 36% (8/22); T3 23% (5/22); T4a 14% (3/22); T4b 9% (2/22); N0 18% (4/22); N1 5% (1/22); N2b 50% (11/22); N2c 14% (3/22); and N3 14% (3/22). The clinical stages were as follows: stage II 14% (3/22), stage III 9% (2/22), stage IVA 59% (13/22), and stage IVB 18% (4/22). As HPV-positive tumors are reported to present mostly at an early T stage and advanced nodal stage [10, 11], more p16-positive patients were distributed in Stage IVA (Figure 2(b)) than p16-negative group mainly due to the N stage.

### 3.2. Immunohistochemical Studies

**Figure 1:**
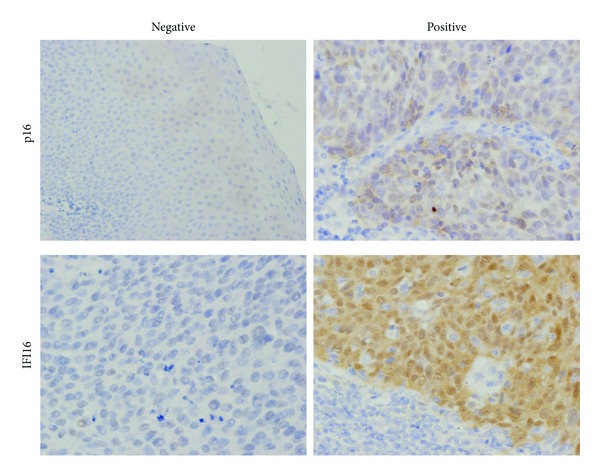
Immunohistochemical analysis of p16 and IFI16 proteins. Examples of immunostaining patterns for the p16 and IFI16 proteins from pretreatment specimens of oropharyngeal SCC.

**Figure 2:**
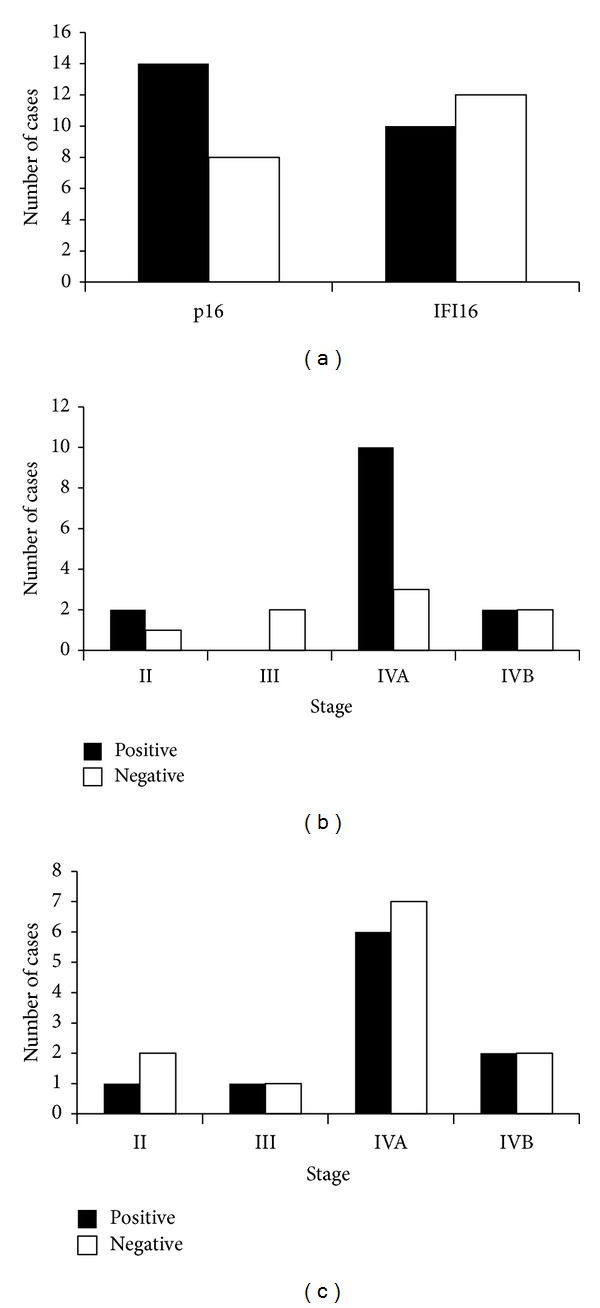
Expression analysis of p16 and IFI16 in all 22 patients. (a) More patients were positive for p16, whereas no difference was observed in IFI16. (b and c) Expression of p16 (b) and IFI16 (c) according to clinical stages. Most of p16 positive patients were stage IVA.

 We used p16 immunohistochemistry (IHC) as a marker of HPV infection, for it is often advocated as a surrogate marker of HPV infection based on the findings that HPV integration with transcription of viral oncoproteins induces the expression of p16 [12, 13]. We examined the expression of p16 and IFI16 protein with the pretreatment specimens of OPSCC (Figure 1). Fourteen of 22 patients (63.6%) were positive for p16 (Figure 2(a)). This positive rate seems to be reasonable for it is reported that about 60% of OPSCC are positive for HPV16 [10]. Ten of 22 (45.5%) patients were positive for IFI16 (Figure 2(a)). In this small number of patients, we could not conclude that the expression of IFI16 is associated with that of p16 (*P* = 0.67).

### 3.3. p16/IFI16 Status and Survival

**Figure 3:**
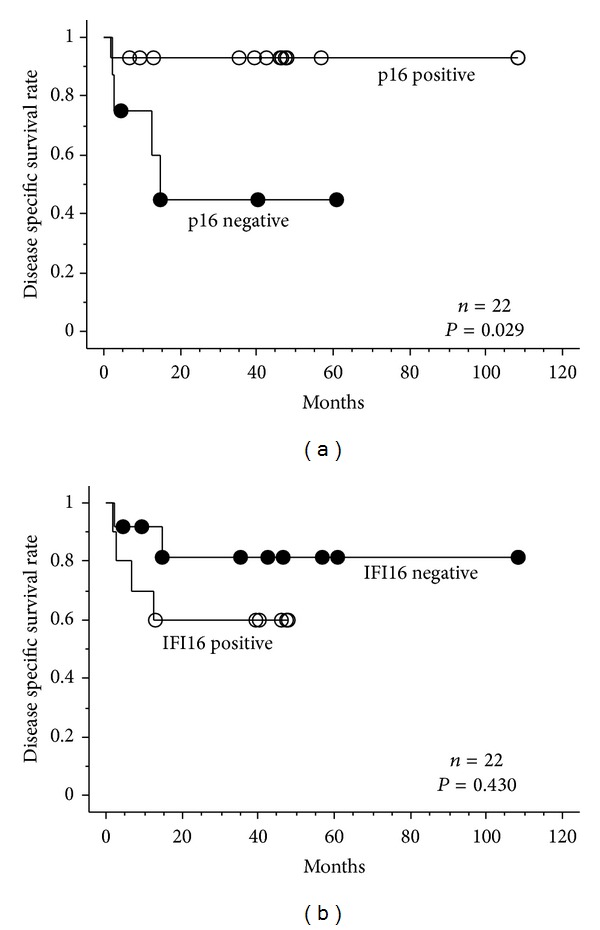
Cumulative prognostic value of 22 patients (Kaplan-Meier analysis). (a) p16 positive patients showed better prognosis (*P* = 0.029). (b) No difference was observed between IFI16 positive and negative patients (*P* = 0.430). Statistical analysis was performed using the log-rank test.

 We calculated the 5-year disease specific survival rate among the OPSCC patients according to p16 and IFI16 status. Compared with the p16 negative group, p16 positive group had a favorable survival rate (*P* = 0.029; Figure 3(a)). On the other hand, there seemed no difference between IFI16 positive and negative groups (*P* = 0.430; Figure 3(b)).

**Figure 4:**
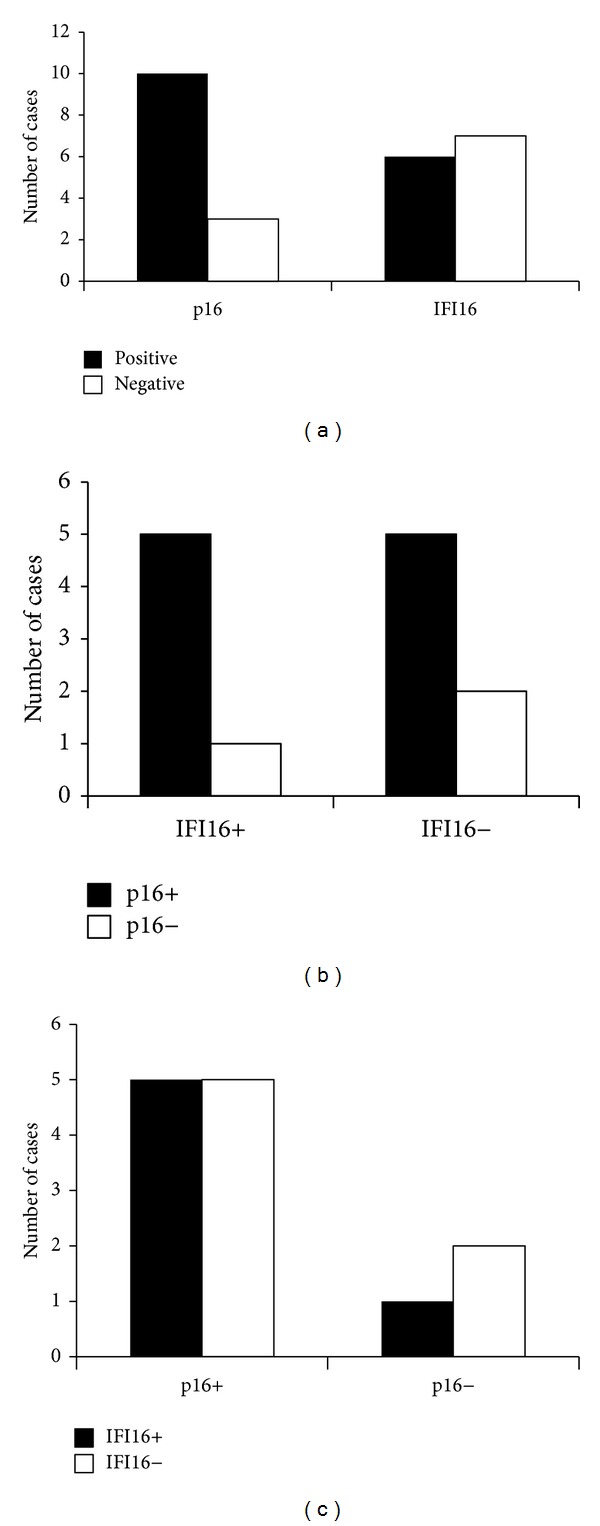
. Expression analysis of p16 and IFI16 in 13 stage IVA patients. (a) More patients were positive for p16, whereas no difference was observed in IFI16. (b and c) Relationship between p16 and IFI16 expression. No correlation was observed between p16 and IFI16 expression. Statistical analyses were performed using the Mann-Whitney *U* test.

**Figure 5:**
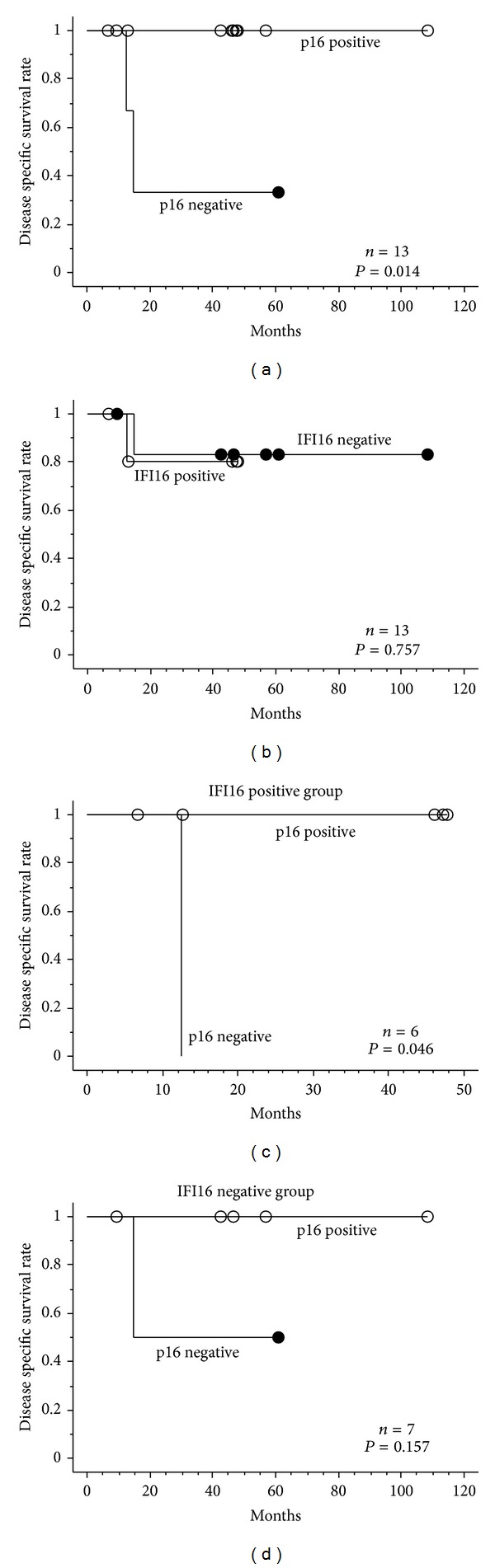
Cumulative prognostic value of stage IVA patients (Kaplan-Meier analysis). (a) p16 positive patients showed better prognosis (*P* = 0.014). (b) No difference was observed between IFI16 positive and negative patients (*P* = 0.757). (c and d) p16 negative patients exhibited worse prognosis in IFI16 positive ((c) *P* = 0.046) group. No significant difference was observed in IFI16 negative ((d) *P* = 0.157) group. Statistical analysis was performed using the log-rank test.

To further analyze the effect of p16 and IFI16 status on the disease outcome, we focused on 13 patients with stage IVA tumors. Ten tumors were positive for p16 (77%) and 6 tumors were positive for IFI16 (46%) (Figure 4(a)). Correlation between p16 and IFI16 expression was not observed (Figures 4(b) and 4(c)). No patient with p16 positive tumor died with the disease, whereas 2 of 3 patients with p16 negative tumor died with the disease (*P* = 0.014; Figure 5(a)). Patients with p16 negative tumor exhibited worse prognosis regardless of whether IFI16 was positive (*P* = 0.046; Figure 5(c)) or negative (nonsignificant, *P* = 0.157; Figure 5(d)). There was no difference in disease specific survival rate between IFI16 positive and negative groups (*P* = 0.757; Figure 5(b)).

## 4. Discussion

In this study, based on the report from Azzimonti et al.,  we hypothesized that HPV related OPSCC expresses IFI16 which regulates tumor progression resulting in favorable prognosis. If IFI16 contributed to the favorable prognosis in HPV related OPSCC, it would be a new predictive biomarker and inducers of IFI16, such as interferon, can be a novel therapeutic arm. However, we were not able to conclude that IFI16 expression is associated with the expression of p16 protein. We used the commercial mouse anti-IFI16 monoclonal antibody which is different from the rabbit polyclonal antibody Azzimonti et al. used [8], so there might be some differences in staining pattern. IFI16 is reported to have tumor suppressive functions in several types of tumors, but there was no difference in the survival rate between IFI16 positive and negative groups in this analysis. One possibility is that IFI16 function was blocked by HPV E6 and E7 proteins. HPV manifests its pathogenesis through E6 and E7 proteins [14]. The E6 protein induces degradation of p53 through ubiquitin-mediated proteolysis, which results in the loss of p53 activity. The E7 protein binds and inactivates the retinoblastoma tumor suppressor gene product pRb, leading to cell proliferation and malignant transformation [1, 10, 14]. IFI16 is reported to manifest its antitumor properties through p53 and pRb proteins. Raffaella et al. showed in vitro that overexpression of IFI16 protein inhibited tube morphogenesis and proliferation of primary endothelial cells but not of HPV16 E6/E7-immortalized cells since IFI16-mediated antiangiogenic activity might depend on the presence of functional p53 and pRb [15]. Contrary to our expectations, in this limited case series, we could not conclude that IFI16 expression is altered in p16 positive OPSCC and that it would be a new predictive marker or a useful therapeutic tool.

## Figures and Tables

**Table 1:** Clinicopathological parameters, p16, and IFI16 detection in 22 oropharyngeal SCC.

Case	Age	Sex	Primary site	T	N	M	Stage	p16	IFI16	Followup, months
1	60	M	PT	1	2c	0	IVA	Neg.	Neg.	NED 60
2	70	M	PT	3	2b	0	IVA	Neg.	Neg.	DWD 14
3	42	M	PT	4a	3	0	IVB	Neg.	Neg.	DWD 2
4	78	M	BOT	3	0	0	III	Neg.	Neg.	NED 4
5	63	M	PT	2	0	0	II	Neg.	Neg.	NED 14
6	55	M	PT	2	2b	0	IVA	Pos.	Neg.	NED 108
7	62	M	PT	2	0	0	II	Pos.	Neg.	NED 108
8	37	M	PT	4a	2b	0	IVA	Pos.	Neg.	NED 56
9	61	M	PT	3	3	0	IVB	Pos.	Neg.	NED 35
10	68	M	PT	3	2b	0	IVA	Pos.	Neg.	NED 46
11	64	M	PT	2	2b	0	IVA	Pos.	Neg.	NED 42
12	49	M	PT	2	2c	0	IVA	Pos.	Neg.	NED 9
13	76	M	PT	4b	3	1	IVB	Neg.	Pos.	DWD 2
14	84	F	PT	4a	2b	0	IVA	Neg.	Pos.	DWD 12
15	55	F	PT	1	1	0	III	Neg.	Pos.	NED 40
16	42	M	PT	1	2b	0	IVA	Pos.	Pos.	NED 47
17	49	M	PT	2	2b	0	IVA	Pos.	Pos.	NED 47
18	55	M	PT	2	2b	0	IVA	Pos.	Pos.	NED 46
19	73	M	PT	3	2c	0	IVA	Pos.	Pos.	NED 6
20	70	M	PT	4b	2b	0	IVB	Pos.	Pos.	DWD 1
21	61	M	BOT	2	0	0	II	Pos.	Pos.	NED 39
22	66	M	PT	1	2b	0	IVA	Pos.	Pos.	NED 12

PT: palatine tonsil; BOT: base of tongue; Neg.: negative; Pos.: positive; NED: no evidence of disease; DWD: died with disease.
